# Risk Characterization in Patients Using Benzodiazepines While Providing Pharmaceutical Care Dispensing Service

**DOI:** 10.3390/pharmacy12040120

**Published:** 2024-07-31

**Authors:** Daida Alberto Armas, Verónica Hernández García, Yanira Román Castillo, Juan Ramón Santana Ayala, Franc Capdevila Finestres, Arturo Hardisson de la Torre, Carmen Rubio Armendáriz

**Affiliations:** 1 Research Group on Environmental Toxicology and Food and Drug Safety, University of La Laguna, Ofra, 38071 Canary Island, Spain; vhernang@ull.edu.es (V.H.G.); farmaciasantanaayala@gmail.com (J.R.S.A.); franc.capde@gmail.com (F.C.F.); atorre@ull.edu.es (A.H.d.l.T.); 2 Nursing Area of the Hospital Nuestra Señora de la Candelaria in Santa Cruz de Tenerife, 38010 Canary Islands, Spain; yanira_ca@hotmail.com

**Keywords:** benzodiazepines, dependence, Tyrer test, community pharmacy, pharmaceutical care

## Abstract

Background: Tolerance and dependence stand out as the most relevant risks observed during benzodiazepine (BZD) treatments. Objectives: To evaluate the degree of dependence of patients on BZD treatments using the Tyrer test; to define a profile of patients at risk of developing BZD dependence; and to discuss the role of the pharmaceutical care offered by the community pharmacy during dispensing. Methods: Prospective cross-sectional descriptive observational study (August 2020–February 2021) involving 127 patients using BZD. They voluntarily answered a questionnaire during the dispensing pharmaceutical care service. The study was evaluated and codified (code: DAA-CLO-2020-01) by the Spanish Agency for Drugs and Health Products (AEMPS), and statistical analysis was performed with SPSS 25.0. Results: 19.05% of patients using BZD were suspected of suffering from BZD tolerance, and 77.88% of all patients were identified as being at a high risk of BZD dependence. The Tyrer test for dependence indicated a mean score of 5.59 out of 13 points. An 18-fold increased risk of developing dependence was detected in the case of coexistence of high anxiety or depression. Conclusions: The community pharmacy, through protocolized care practices and supported by tools such as the Tyrer test, can play a decisive role in the detection, prevention, and resolution of the risks associated with BZD treatments.

## 1. Introduction

Benzodiazepines (BZDs) have been used clinically for more than 50 years to treat disorders such as insomnia, anxiety, and epilepsy, as well as to aid muscle relaxation and anesthesia [1,2]. These drugs are the main group of sedative–hypnotics currently prescribed [3,4,5,6,7]. Some studies have characterized the most common BZD profile of users as female, socially isolated, and with a history of substance abuse [8,9].

When BZD treatments are not aligned and exceed the recommendations suggested by clinical practice guidelines and technical data sheets in relation to doses and duration, adverse effects and health risks like cognitive impairment, tolerance, and dependence, among others, may occur [10,11,12]. BZD usefulness is limited by the development of either or both tolerance to most of their pharmacological actions and dependence [1,2,13], which is a special form of addiction [14].

Dependence on BZDs has been associated not only with the pharmacokinetic characteristics of BZDs but also with several patient and treatment characteristics [15]. The risk of developing physical and psychological dependence increases with the dose of BZDs (although it also occurs at low doses) and the duration of the BZD treatment (if longer than 2 weeks), and shorter the half-life, and higher the potency of BZDs. Low-dose prescriptions and intermittent administration do not ensure that dependence does not occur but may, at least, contribute to reducing its magnitude [16]. Pharmaceutical innovation has been committed to prevent BZD dependence by developing less-addictive BZDs [17,18]. Although different ways of measuring the degree of dependence have been established, one of the most widely used validated tests for primary care was developed by Tyrer [19].

BZD dependence may generate withdrawal syndrome, a condition that is often difficult to differentiate as it is confused with a relapse of the original anxious condition [20,21]. Depending on the degree of withdrawal symptoms, two situations may occur: minor withdrawal syndrome (anxiety, insomnia, depersonalization, sensory disturbances, and somatic symptoms: palpitations, sweating, fatigue, nausea, hyperventilation, irritable bowel, and loss of appetite, among others) and major withdrawal syndrome (delirium, hallucinations, confusion, and convulsions, among others) [16].

BZD tolerance develops at different rates depending on the pharmacological action, suggesting the existence of distinct mechanisms for each behavioral parameter. Because most of the pharmacological actions of BZDs are mediated through GABAA receptor binding, adaptive alterations in the number, structure, and/or functions of these receptors may play an important role in the development of tolerance [1]. The prolonged activation of GABAA receptors by endogenous and exogenous modulators induces adaptive changes that lead to tolerance [2]. Tolerance develops to hypnotic effects within days to weeks, to myorelaxant effects within weeks, to anticonvulsant effects within weeks to months, and to anxiolytic effects within months. This explains why patients commonly increase dosage over time and many eventually take more than one type of BZDs after the first loses effectiveness [22].

Spain presents a worrying picture regarding the use of psychoactive medicines, with a notable increase in recent years. Between 2000 and 2012, the consumption of anxiolytics and hypnotics in Spain increased by 46.8% and 81.8%, respectively [23]. In 2021, prescriptions for anxiolytics and antidepressants in Spain increased from 90,603 to 93,046 DDD (Defined Daily Doses per 1000 inhabitants per day), representing an increase of 2.7% [23]. According to The International Narcotics Control Board (INCB), Spain ranks first in the world in benzodiazepine consumption, with 110 DDD per 1000 inhabitants per day in 2020. In 2021, Spain reached 93.30 daily doses of benzodiazepines per 1000 inhabitants, an increase of 10 percentage points since 2010. This figure places Spain at the top of the world ranking for benzodiazepine use, surpassing even countries such as Belgium and Portugal, which are in second and third place, respectively [24].

The role of community pharmacy nowadays could not be understood without the provision of pharmaceutical care, “the active participation of the pharmacist in ensuring improved patient quality of life, through dispensing, over the counter (OTC) prescription and pharmacotherapy follow-up. Such participation implies cooperation with the physician and other healthcare professionals in order to secure outcomes that improve patient quality of life, as well as pharmacist intervention in activities that offer good health and avoid the development of diseases” [25].

There are wide-ranging and disparate levels to support the further development community pharmacy (CP) pharmaceutical care (PC) services like dispensing [26]. The dispensing service was defined by the Spanish Forum on PC-CP (2024) [25] as “the professional service of the pharmacist destined to ensure—following individual evaluation—that the patients receive and use their medicines adequately in relation to their clinical needs, at the precise doses indicated for their individual necessities, during the adequate period of time, with information for correct use, and in abidance with current legislation”.

When attempting to implement pharmaceutical care services to drive change, community pharmacies face numerous challenges [27,28,29,30]. These include aligning funding with desired services, undergraduate educators and professional leaders setting expectations for the pharmacists’ role in practice, and the availability of sufficient funding and time for both specific extended service accreditation and broader postgraduate training [26].

Taking into account the above, the main purpose of this study is to assess the degree of dependence of patients on BZD treatment using the Tyrer test. In addition, the profile of patients at risk of developing BZD dependence and the potential role of the pharmaceutical care dispensing service offered in the community pharmacy were established as secondary aims.

## 2. Materials and Methods

(a) Study design

Prospective cross-sectional observational study with a single-center analytical component carried out for 6 months (August 2020–February 2021).

(b) Study setting

The study setting was a community pharmacy in Tenerife (Canary Islands, Spain). The study was evaluated and codified (code: DAA-CLO-2020-01) by the Spanish Agency for Drugs and Health Products (AEMPS).

(c) Study population

A total of 127 patients of both sexes between 18 and 90 years of age with a prescription for one of the ten BZD drugs under study (lorazepam, lormetazepam, alprazolam, diazepam, bromazepam, clorazepate potassium, clonazepam, ketazolam, clobazam, and flurazepam) were interviewed during the dispensing pharmaceutical care service. The sample size corresponds to an acceptable level with a confidence interval of 95% and an estimated precision of 5%. Patient participation was voluntary, and an informed consent form was signed.

(d) Inclusion and exclusion criteria

Inclusion criteria: patients starting or continuing treatment with BZD as a monodrug therapy (lorazepam, lormetazepam, alprazolam, diazepam, bromazepam, clorazepate potassium, clonazepam, ketazolam, clobazam, and flurazepam); patients aged between eighteen and ninety years of age; patients who agreed to voluntarily participate in the study and who signed the informed consent form; patients whose communication and/or decision-making abilities were not impaired; caregivers who go to the pharmacy to pick up a BZD prescribed for the patient they care for (a caregiver is defined as a person who is responsible for the acquisition and administration of medication for a dependent patient, whether or not they are a relative).

Exclusion criteria: patients who, although fulfilling the inclusion criteria, did not agree to participate in the study; patients who did not agree to sign the informed consent form; patients prescribed with combinations of BZDs or other active ingredients; patients not evaluable for a variety of reasons, at the discretion of the researcher, including incomplete records, suspicion of transcription errors in the database, and unverified suspicion of exclusion criteria, among others; patients with communication, psychological, or linguistic difficulties or without decision-making capacity; patients who decided to leave the study voluntarily; pregnant or breastfeeding women; patients referred from other professional pharmaceutical care services, as they may skew the results, given that patients would have received personalized information about their medication in each of these services, so their knowledge may be greater than that of patients who have not received this information and the data may contain a bias.

(e) Study tool

Data collection was carried out by means of a structured clinical interview during the BZD dispensing pharmaceutical care service. The data collection questionnaire included the Tyrer test for BZD dependence along with other variables like gender, age, years on BZD treatment, number of drugs prescribed simultaneously with BZD (monotherapy; 1–4 drugs and >5 drugs), additional active treatments known for interacting with BZD, reading the package leaflet, knowledge of side effects, occurrence of body falls, pain/discomfort and anxiety/depression (dimension 4 and 5 of the validated EuroQol 5D-3L questionnaire [31,32], and the Pfeiffer test for cognitive impairment [33]).

Tyrer dependence test for BZD dependence (Table 1) is made up of 6 items in which the corresponding score is assigned according to each patient and a degree of “no dependence”, a “certain degree of dependence”, a “high risk of dependence”, and “present dependence” on a gradual scale (0–13). Tolerance to BZDs was detected using a question present in the questionnaire that investigated if patients needed a higher dose of BZDs to achieve the same effects as those obtained when the BZD treatment was initiated [34].

The statistical analysis consisted, firstly, of a description of the participants, including 95% confidence intervals (CIs) and, secondly, of a correlation study between the different degrees of dependence: “high” and “present” (the category “some degree” was suppressed as it did not have a representative number of patients for statistical analysis).

For the processing of the Tyrer test data, the numerical scale variable is explored using the Kolmogorov–Smirnov test at a significance level of *p* ≤ 0.05 to check whether or not it follows a normal distribution. It is concluded with non-parametric tests that this variable does not follow a normal distribution.

The Pfeiffer questionnaire for cognitive impairment (Short Portable Mental Status Questionnaire (SPMSQ)) is excellent for geriatric, primary, and specialized care. It is composed of 9 questions and is scored on errors rather than on correct answers, with variation in patients with a low level of education [33] (Table 2).

Measuring quality of life requires the use of a questionnaire to assess the patient’s perception in a quick and simple way. The EuroQol 5D-3L (Table 3) is a validated generic instrument that can be used in both healthy individuals and groups of patients with different pathologies [32]. The patient assesses his/her health status, first at the levels of severity composed of 5 health dimensions, i.e., ease of mobility, ease of self-care, ease of daily activities, presence of pain and/or discomfort, and possible states of anxiety and/or depression, and each of them has three levels of severity, i.e., no problems, moderate problems, and severe problems, which provide the information of the study [31].

(f) Statistical analysis

All variables were processed with mean comparative tests (Pearson’s chi square and ANOVA) considering a significance of *p* < 0.05. Finally, a multivariate binary logistic regression model adjustment (Wald criterion) was performed with the degree of dependence on BZDs as an effect to estimate the risk of “present dependence” jointly for all factors with a significance of *p* ≤ 0.05.

Data analysis was performed using SPSS 25.0™ from IBM Co.^®^ (Armonk, NY, USA) on a Windows NT 365 Professional™ operating system from Microsoft Co.^®^ (Redmond, WA, USA).

## 3. Results and Discussion

The sample (127 users of BZDs) consisted of 66.14% women and 33.86% men, with a mean age of 61 years and treated with BZDs for an average of 6.5 years. This long duration of the BZD treatment reveals its lack of safety and misuse and could be identified as a risk to be address by all healthcare providers.

Table 4 shows the percentages for the different types of benzodiazepines (BZDs) studied. It is important to note that a patient may be prescribed more than one BZD, which explains why the total number of BZDs amounts to 153 molecules.

While 80.95% of total patients consider that they do not need more than the prescribed dose to achieve the therapeutic objective (it is noteworthy that the total number of responses obtained for tolerance was 126 as one patient did not answer this question), 19,05% of BZD users could be suffering from BZD tolerance since they report needing a higher dose of BZDs to achieve the same effects.

**Figure 1:**
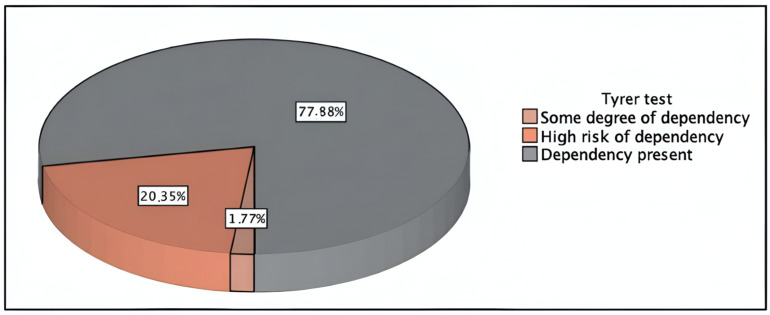
Risk of BZD dependence in BZD users according to the Tyrer test.

Table 5 shows the results of the Tyrer test (mean: 5.59; SD: 2.62). Overall, 77.88% of patients using BZDs have a high risk of developing dependence on these drugs, and 20.35% are already dependent on BZDs (Figure 1). The relative frequency of patients with BZD dependence at the 95% CI ranges from 70.65% to 85.35%. Considering that one of the parameters that the Tyrer test includes is “BZD treatment duration > 3 months” and most of our patients comply with this (the average treatment time is 6.5 years), these results align with the study expectations. Previous studies by Minaya et al. [8] and Gómez et al. [34] observed 69.2% dependence among BZD users, with 92.6% of the population showing withdrawal symptoms.

The characterization of patients with BZD dependence during the dispensing pharmaceutical care service is an innovative approach of this study that supports the further development of community pharmacy services like dispensing as stated by Morris et al. [26], Schommer et al. [35], and Gastelorrutia et al. [36]. Patients with BZD dependence should be considered targets for personalized pharmaceutical interventions (PIs) during the dispensing service and candidates for the follow-up PC service.

Table 6 details the correlations between the different degrees of dependence (“high” and “present”) and other variables like gender, age, years of BZD treatment, number of drugs prescribed simultaneously with the analyzed BZDs, other active treatments with interactions with BZDs, patients reading the package leaflet, patients’ knowledge of side effects, patients’ body falls, presence of pain/discomfort, occurrence of anxiety/depression according to the EuroQol 5D-3L, and cognitive disorders (positive or negative) according to the Pfeiffer test.

When analyzing the correlation between BZD dependence and gender, women show higher dependence levels than men. “High BZD dependence” has reached 62.5% in women compared to 37.5% in men. “Present dependence” to BZD was detected in 82.6% of women compared to 17.4% of men. Although this apparent gender inequality does not reach statistical significance, Villa et al. [37] also reported higher dependence in women.

De la Cruz et al. [38] studied hidden addictions in women from the Canary Islands and detected BZD dependence in 44.52%. These results from 2008 show a lower level of dependence as both the methodology followed by these authors and the timing were different from ours. The differences between the two studies may be influenced not only by the characteristics of the patients and prescribing patterns of the healthcare providers but also by an upward trend of the use of anxiolytics and hypnotics in Spain as reflected in the data from 2020 [23].

In relation to age and dependence, the results show that being older than 64 ± 11.7 years involves a higher risk of BZD dependence compared to patients aged 59 ± 15.4 years. Since statistical tests do not confirm this correlation (no statistical difference was observed), age was not identified as a risk factor for BZD dependence among our patients.

The duration of the BZD treatment was another factor whose correlation with BZD dependence was studied. Although no statistical significance difference was detected, the results with a 95% CI indicate that patients with “present BZD dependence” have been in treatment for longer (8.9 ± 8.9 years) than patients with a “high risk of dependence” (5.7 ± 6.6 years). Previously, Villa et al. [37] measured the dependence according to the DSM-IV criteria [39] and determined that 28% of people who use psychotropic drugs develop dependence and, when counting those who have been in treatment for more than one year, this proportion rises to 37.9% of patients.

Polymedication was another factor correlated with BZD dependence. Although we hypothesized that the simultaneous use of a higher number of drugs was related to a higher degree of dependence on BZDs, no statistical significance was obtained. Nevertheless, our results highlight that while 47.7% of patients that show a high risk of dependence use more than five different drugs, 73.9% of BZD users with “present BZD dependence” also use more than five different medications. Another risk derived from polymedication is derived from drug–drug interactions. Villa et al. [37] reported 40% BZD dependence among patients using a combination of BZDs and antidepressants/anxiolytics. The occurrence of drug–drug interactions and their correlation with BZD dependence was studied, and results showed that patients with “present BZD dependence” experience more drug–drug interactions (95.65%). Therefore, patients with the highest number of drug–drug interactions are also those with the highest BZD dependence. This is demonstrated by a statistical significance of *p* < 0.05. This result points out an unsafe situation that could be prevented and detected either by the prescribing physician of the dispensing pharmacist.

Multivariate statistical analysis also showed that when a patient using BZD switches from monotherapy to the use of 2–4 drugs or more than 5 drugs, the risk of BZD dependence increases by 4.8 times (OR: 4.8; 95% CI: 1.4–16.2). Our assessment concludes that the presence of factors associated with polymedication such as the chronicity of treatment and advanced age (considered for the study as over 65 years of age) are characteristics observed in patients with BZD dependence. Therefore, these BZD users have been identified as a potential group of patients to whom personalized BZD dispensing pharmaceutical care service should be offered with the aim of promoting safe and effective BZD use.

In order to investigate if the patient’s knowledge about the BZD consumed is a relevant factor for the occurrence of BZD dependence, the frequencies of the different types of BZD dependence and the attitude of the patients towards the reading of the BZD package leaflet were correlated. The proportions of both categories of dependence among the patients who declared to have read the leaflet (56.8% “high” versus 60.9% “present”) do not show striking differences, a fact that is confirmed by the absence of statistical significance. Patients who have not read the leaflet, on the other hand, show a high dependence of 43.2% and a current dependence of 39.1%. However, these results provide information on the opportunity of improving the patients’ knowledge (health education) of BZDs not only during the dispensing pharmaceutical service but also in the process from prescribing BZDs to the end of the treatment.

When the patients’ awareness of the side effects was correlated with the occurrence of BZD dependence, an unusual association was observed. While 87% of patients with “present BZD dependence” declared to know the BZD side effects, only 60.2% of BZD users with a “high risk of BZD dependence” admitted of having the knowledge of BZD side effects. It may be that patients with “present BZD dependence”, having been treated with BZDs for more years, have more experience and know more about their side effects, as they have been more likely to suffer them. These assumptions are confirmed by the statistical significance (*p* < 0.05).

The higher prevalence of body falls among BZD users was also selected as a health risk to be correlated with the occurrence of BZD dependence. Nevertheless, our assessment confirmed a lack of association between BZD dependence and body falls based on the non-significance of the differences shown by the statistical test applied in the comparisons.

The presence of pain or discomfort was also correlated with the occurrence of BZD dependence. The highest prevalence of both dependence categories (62.5% of high risk of BZD dependence and 43.5% of present BZD dependence) was observed among the patients using BZDs who did not suffer any pain or discomfort. As pain and discomfort increase, the dependence on BZDs decreases, and this may be because patients with a high degree of pain are concomitantly prescribed additional analgesic treatments, such as opioids [39]. However, these findings could not be confirmed with statistical significance.

Patients identified as being at a “high risk of BZD dependence” showed “no anxiety or depression” (47.7%), “moderate anxiety or depression” (46.6%), and “high anxiety or depression” (5.7%). But these figures were higher when patients with “present BZD dependence” were evaluated. When BZD dependence was already established in patients, 39.1% of patients were also moderately depressed and 47.8% were very depressed. These findings were confirmed by the statistical significance (*p* < 0.001). Perhaps a possible justification for the results lies in the degree of tolerance to BZDs. Patients with “present BZD dependence” have been on BZD treatment for more years, and during this time, they have developed greater tolerance to BZD treatment, and consequently continue to manifest health problems such as anxiety and depression, among others.

The results of the multivariable binary logistic regression model showed that a state of anxiety or depression rated by the patient as extreme (“I am very anxious or depressed”) increases the likelihood of present BZD dependence by 18 times (OR: 18; 95% CI: 3–96) compared to that of patients who report no anxiety or depression (“I am not anxious or depressed”). The patients who reported a state of moderate anxiety or depression did not display an increased risk of BZD dependence. This result was predictable and supports our hypothesis of the opportunity of enhancing the pharmaceutical care services in patients with extreme anxiety and depression and who request the dispensing of BZDs in order to minimize the health risks derived from these drugs.

In patients with no cognitive impairment according to the Pfeiffer test, high percentages of BZD dependence stand out, both for present BZD dependence (73.9%) and for high risk of BZD dependence (88.6%). On the other hand, in patients with positive cognitive impairment according to the Pfeiffer test, the prevalence of “present BZD dependence” only reached 26.09%. These results point out that no relationship between cognitive impairment and patient dependence on BZDs could be established. The non-significance of the differences obtained with the statistical test used in the comparison confirms this observation.

In view of the results obtained with the OR and with the aim of transferring these results to the healthcare practice, we could suggest that, without the need to administer the BZD dependence test (the Tyrer test), any patient requesting the dispensing of a BZD and who also presents a strong state of anxiety or depression and has been prescribed more than one drug is a candidate for receiving a personalized pharmaceutical care service to detect, prevent, and mitigate the risk of BZD dependence.

Finally, according to the results, the following two profiles of patients with BZD dependence have been characterized:

Patients with present BZD dependence: generally detected in 65-year-old women with an average of 8.9 years of treatment with BZDs, polymedicated, and with drug interactions. In general, these BZD users are moderately to very depressed or anxious and present a negative cognitive impairment test result (the Pfeiffer test). Overall, 50% of patients with this profile read the BZD leaflet and are aware of the side effects of the BZD medication and show no registry of body falls, nor associated pain or discomfort.

Patients with a high risk of BZD dependence: generally younger women (59-year-old) with a mean number of years on BZD treatment of 5.7. They are following a polymedicated treatment (44.3% take 1–4 medications, and 47.7% take more than 5 medications), presenting a significant prevalence (63.3%) of drug–drug interactions. They are not depressed or anxious or are only mildly depressed and, in general, they show negative cognitive impairment according to the Tyrer test. Half of the patients have read the BZD package leaflet and are aware of the BZD side effects. These patients have not suffered previous body falls and do not report pain or discomfort.

In the case of community pharmacies, there is a real opportunity for providing a personalized Pharmaceutical Care according to the profile of these at-risk patients using BZDs. Moreover, the promoting of the pharmacist-patient bond through pharmaceutical services could improve not only the safety and effectiveness of the BZD treatment but the patient’s quality of life.

Limitations: One of the limitations of the study may be related to the final sample size. Nevertheless, as detailed in the statistical procedure, we have managed to maintain statistical significance. Furthermore, all cross-sectional studies have some limitations, especially when compared with the cohort or case–control studies that compare results over a period of time. However, it is worth noting the strength of cross-sectional studies in terms of time and minimal cost.

Strengths: This study lies in the specific focus and choice of a significant problem, which is relevant to both public health and pharmacy practice, such as benzodiazepines (BZDs) and their associated tolerance and dependence. It is underpinned by a robust methodology, with a sizeable sample and data collected in a real clinical setting, enhancing the external validity of the study. Using a validated tool such as the Tyrer test to measure dependence provides quantifiable and standardized results. Assessment and coding by AEMPS add credibility and ensure that the study meets the necessary ethical and methodological standards. The significant results regarding the high risk of dependence underline the importance of the problem, which may have a considerable practical impact on healthcare.

## 4. Conclusions

The degree of dependence among patients using BZDs is high and associated with different factors related to the characteristics of the treatment and the patient. Patients taking polymedication have a statistically higher risk of BZD dependence. No relationship between cognitive impairment and patient dependence on BZDs could be established. There is an important opportunity to enhance pharmaceutical care services for patients with extreme anxiety and depression who request the dispensing of BZDs to minimize the health risks associated with these drugs. The prevalence of BZD dependence highlights the potential of every healthcare provider to prevent, detect, and minimize their health risks. Community pharmacy, through protocolized collaborative care practices and with the support of measurement tools such as the Tyrer test, can play a decisive role in the detection, prevention, and resolution of this dependence. The health education of patients with a profile susceptible to BZD dependence along with communication and collaborative work of healthcare teams are complementary measures to minimize the risks associated with BZD treatments.

## Figures and Tables

**Table 1:** Tyrer test for BZD dependence [19].

BZD Dependence Test	Score
1. BZD	3
2. High doses (above average)	2
3. Duration of treatment longer than 3 months	2
4. Drug- or alcohol-dependent personality or history of drug or alcohol dependence	2
5. Short half-life of BZD	2
6. Evidence of tolerance or dose escalation	2
TOTAL SCORE	

0: no dependence; 1–4: some degree of dependence on BZDs; 5–8: a high risk of dependence on BZDs; 8–13: the presence of BZD dependence.

**Table 2:** Validated Pfeiffer test for cognitive impairment [33].

Pfeiffer Test	Mistakes
1. What day is today—day, month, year?	
2. What day of the week is today?	
3. Where are we now?	
4. What is your telephone number?	
5. What is your address? (only if you do not have a telephone)	
6. How old are you?	
7. What is your date of birth—day, month, year?	
8. Who is now the President of the Government?	
9. What are your mother’s two surnames?	
TOTAL SCORE	

Positive cognitive impairment > 3 errors (4 illiterates); negative cognitive impairment < 3 errors.

**Table 3:** Validated EuroQol 5D-3L test for quality-of-life assessment [31].

EuroQol 5D-3L Test
1. MOBILITY - I have no problems walking. - I have some problems walking. - I have to stay in bed.
2. PERSONAL CARE - I have no problems with personal care. - I have some problems with washing or dressing myself. - I am unable to wash or dress myself.
3. DAILY ACTIVITIES - I have no problems in carrying out my daily activities. - I have some problems in carrying out my daily activities. - I am unable to carry out my daily activities.
4. PAIN/DISCOMFORT - I have no pain or discomfort. - I have moderate pain or discomfort. - I have a lot of pain or discomfort.
5. ANXIETY/DEPRESSION - I am neither anxious nor depressed. - I am moderately anxious or depressed. - I am very anxious or depressed.

**Table 4:** Types of BZDs prescribed to the study patients.

BZD-Type Frequencies				
		Answers		Percentage of Cases
		Nº	Percentage	
Benzodiazepines	lorazepam	39	25.5%	30.7%
	lormetazepam	22	14.4%	17.3%
	alprazolam	21	13.7%	16.5%
	diazepam	22	14.4%	17.3%
	bromazepam	13	8.5%	10.2%
	clorazepate	19	12.4%	15.0%
	clonazepam	9	5.9%	7.1%
	ketazolam	3	2.0%	2.4%
	clobazam	2	1.3%	1.6%
	flurazepam	3	2.0%	2.4%
Score		153	100.0%	120.5%

**Table 5:** Statistical analysis of Tyrer BZD dependence test in patients using BZD.

		Tyrer BZD Dependence Test Score
N		127
Normal parameters	Mean	5.59
	Standard deviation (SD)	2.623
More extreme differences	Absolute	0.285
	Positive	0.219
	Negative	−0.285
Z of Kolmogorov–Smirnov		3.211
Asymptotic significance (bilateral)		0.000

**Table 6:** Correlation between the degree of BZD dependence (Tyrer test) and the various variables under study.

Factor Explored	Degree of Dependence on BZDs (Tyrer Test)		*p*-Value
	High	Present	
Gender:			0.070
Men	37.5%	17.4%	
Women	62.5%	82.6%	
Age (years)	59.0 ± 15.4	64.4 ± 11.7	0.123
Years in treatment	5.7 ± 6.6	8.9 ± 8.9	0.054
Number of drugs:			0.059
Monotherapy	8%	0%	
From 1–4	44.3%	26.1%	
>5	47.7%	73.9%	
Medications interacting with BZDs:			0.002
Yes	63.6%	95.7%	
No	36.4%	4.3%	
Leaflet reading:			0.815
Yes	56.8%	60.9%	
No	43.2%	39.1%	
Knowledge of side effects:			0.025
Yes	60.2%	87%	
No	39.8%	13%	
Body falls:			0.509
Yes	12.5%	17.4%	
No	87.5%	82.6%	
Pain/discomfort:			0.171
No pain/discomfort	62.5%	43.5%	
Moderate pain/discomfort	25%	30.4%	
Very pain/discomfort	12.5%	26.1%	
Anxiety/depression:			<0.001
No anxiety/depression	47.7%	13%	
Moderate anxiety/depression	46.6%	39.1%	
Very high anxiety/depression	5.7%	47.8%	
Pfeiffer test:			0.095
Positive cognitive impairment	11.4%	26.1%	
Negative cognitive impairment	88.6%	73.9%	

## Data Availability

No new data were created or analyzed in this study. Data sharing is not applicable to this article.
